# COVID-19 education policy often relied on older research despite available new evidence

**DOI:** 10.1016/j.xpro.2026.104710

**Published:** 2026-07-24

**Authors:** Basil Mahfouz, Licia Capra, Geoff Mulgan

**Affiliations:** 1 Department of Science, Technology, Engineering and Public Policy (STEaPP), UCL, London, UK; 2 Department of Computer Science, UCL, London, UK

**Keywords:** Health Sciences, Systems biology, Computer sciences

## Abstract

Policymakers are increasingly relying on scientific research, but it remains unclear whether policy decisions reflect the latest available evidence. Building on a previously developed semantic matching approach, we use pretrained language-model embeddings to identify newer studies that are substantively related to research cited in policy documents. We then examine whether these related studies were already available before the policy documents were published. Applied to COVID-19 education policy, the analysis shows that policy documents often continued to cite established pre-pandemic research even when similar post-2020 studies had already been published. This pattern suggests that policy decisions do not always keep pace with newly available research. The approach provides a scalable way to diagnose how quickly policy systems incorporate emerging evidence across domains.

## Highlights

• COVID-19 education policies often cited pre-pandemic research
• 56% of older cited studies had newer related evidence available
• Older cited papers had higher citation impact and more news coverage
• Semantic matching reveals update latency in policy evidence use

## Graphical abstract

**Figure:**
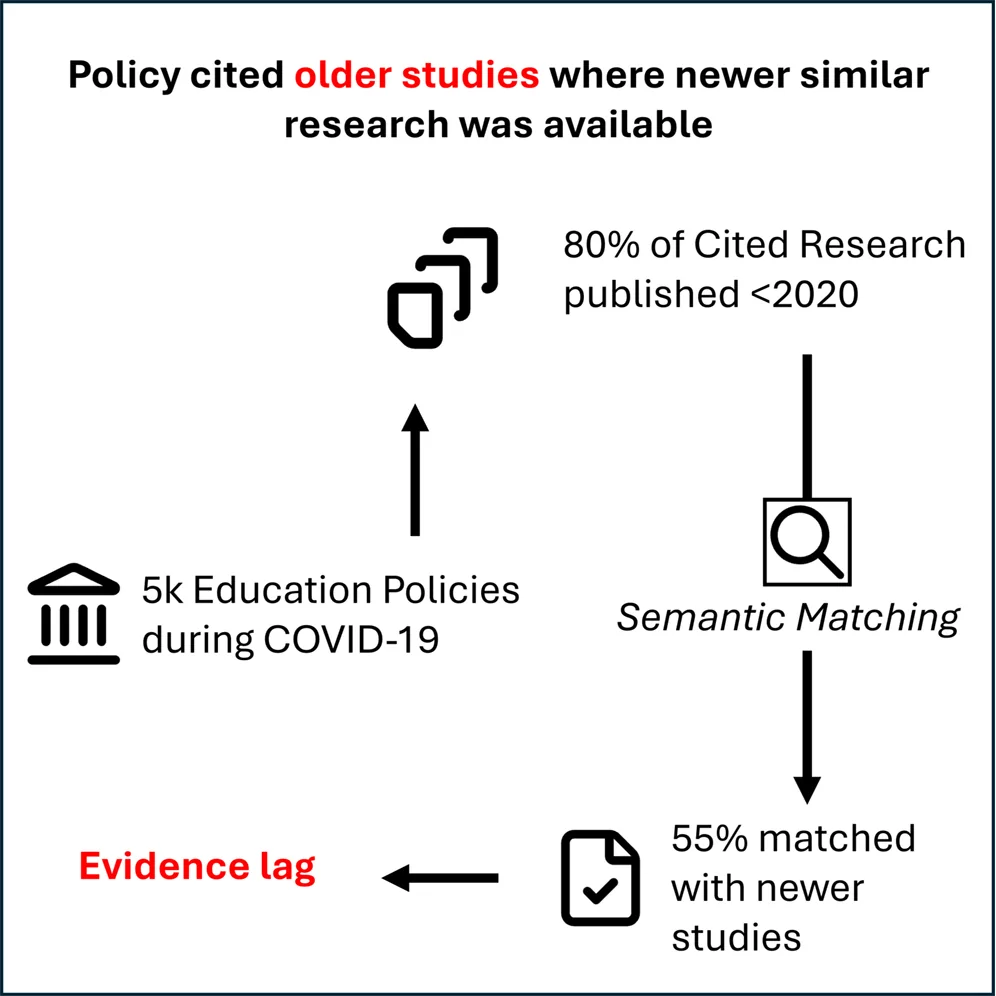

## Introduction

Policymakers often rely on scientific research to inform public decisions. Yet it remains unclear to what extent policy decisions keep pace with newly available evidence. This question becomes particularly important in crisis situations, when scientific knowledge evolves rapidly while governments must act under conditions of urgency and uncertainty.

The COVID-19 pandemic provides a unique case study of evidence-use systems. Education policy faced acute pressure as governments decided when to close schools, how to reopen them, whether to adopt remote or hybrid learning, and which mitigation measures to implement, often under severe time constraints and with substantial social consequences. At the same time, scientific activity rapidly reoriented toward the pandemic, generating a large and fast-growing body of education research aimed at informing policy responses.^1^^,^^2^^,^^3^^,^^4^^,^^5^ This combination of urgent decision-making and rapid research production makes COVID-19 education policy a unique case for examining evidence use under crisis conditions.

Empirical studies of science use during the pandemic present contrasting interpretations. Qualitative research and practitioner accounts consistently describe persistent barriers to evidence use, including institutional routines, limited analytical capacity, and weak alignment between academic outputs and policy needs.^6^^,^^7^^,^^8^^,^^9^ By contrast, large-scale analyses of policy citations report frequent references to recent and highly visible COVID-19 research, suggesting closer temporal alignment between scientific production and policy activity.^10^^,^^11^

A key limitation underlies much of this quantitative evidence. Citation-based analyses treat uncited research as a single residual category, implicitly assuming that uncited papers were plausible candidates for policy use.^12^^,^^13^^,^^14^^,^^15^^,^^16^ In practice, this assumption is unlikely to hold. Academic research varies widely in its relevance to policy questions and in its timing relative to decision-making needs.^17^^,^^18^^,^^19^ As a result, citation-based measures can conflate two distinct cases: where relevant research did not yet exist, and where it existed but was not incorporated. This limits what policy citations alone can reveal about responsiveness to newly available evidence.

This limitation reflects a broader problem of contextual interpretation. Noyons’ Area-Based Connectedness framework argues that science–society linkages should be interpreted relative to the research areas in which they are embedded rather than through isolated indicators alone.^20^ Applied to policy citations, this implies that references cannot be interpreted independently of the surrounding body of research addressing the same topic. Research production varies widely across areas: some fields generate large volumes of policy-relevant work, while others produce relatively little, and the institutional pathways linking research and policy also differ across domains. Without considering the surrounding research landscape, citation-based measures cannot distinguish between cases where relevant research did not exist and cases where relevant research existed but was not incorporated into policy.

Determining systematically which publications were substantively relevant to a specific policy question at a particular moment is difficult. One strategy is to anchor the analysis in research that policy demonstrably used and then examine nearby alternatives. Marx and Hsu’s “twinning” method provides one way to implement this logic by identifying independent papers reporting the same or highly similar scientific discovery. Because these twin papers represent the same underlying advance, comparing them allows researchers to examine differences in downstream outcomes while holding the scientific contribution constant.^21^ However, identifying twin discoveries in their study required manual matching, which limits the approach to smaller datasets.

Recent advances in natural language processing made it possible to scale this twinning logic. Mahfouz et al. implement the approach using pretrained language models to identify semantically similar research across large publication corpora without manual classification.^19^ Although automated matching is less precise than manually identifying twin discoveries, it enables the approach to be applied across millions of publications and has already been applied in various contexts.^22^

In their application, substantively similar papers published within the same time frame are paired and compared to examine which research subsequently becomes cited in policy while holding research content approximately constant. In this study, we apply the same scalable twinning framework to a different question. Rather than examining why some papers become cited while others do not, we use semantic matching to examine whether policy incorporated the most recent available research.

For each paper cited in a policy document, we identify substantively similar studies that were published later but before the policy document was issued. These papers represent plausible newer alternatives that could have informed the same policy discussion. We apply this framework to COVID-19 education policy by examining policy documents that cite research published before the pandemic and assessing whether substantively similar studies were published during the surge in pandemic-era research before those policies were issued.

We also compare cited papers and their newer semantically similar counterparts across bibliometric and altmetric indicators associated with policy uptake. Specifically, we examine Field-Weighted Citation Impact (FWCI), a field-normalised measure of scientific influence; journal CiteScore, a proxy for journal visibility and prestige; first-author h-index at the time of publication, capturing prior author productivity and academic impact; and news mentions, reflecting public visibility and media attention. These comparisons allow us to assess whether continued reliance on older research may reflect differences in academic visibility, journal prestige, author prominence, or public attention, rather than timing alone.

By introducing this temporal dimension, the analysis distinguishes between the availability of relevant research and its incorporation into policy. Conditioning on both semantic similarity and timing allows us to test whether policy relied on older evidence even when closely related and more recent research were already available, separating reliance driven by the absence of research from reliance shaped by the functioning of policy evidence-use systems.

## Results

Our query of the Overton database identified a global corpus of more than 5,000 COVID-19 education policy documents issued between 2020 and 2023. Across the scholarly references extracted from these documents, 80% of the cited Scopus-indexed papers (5,541 articles) were published before March 2020. More than half of these pre-2020 studies (3,113 papers) could be matched to at least one post-2020 publication exceeding the semantic similarity threshold (cosine ≥ 0.8) and published at least three months before the citing policy document.

**Figure 1:**
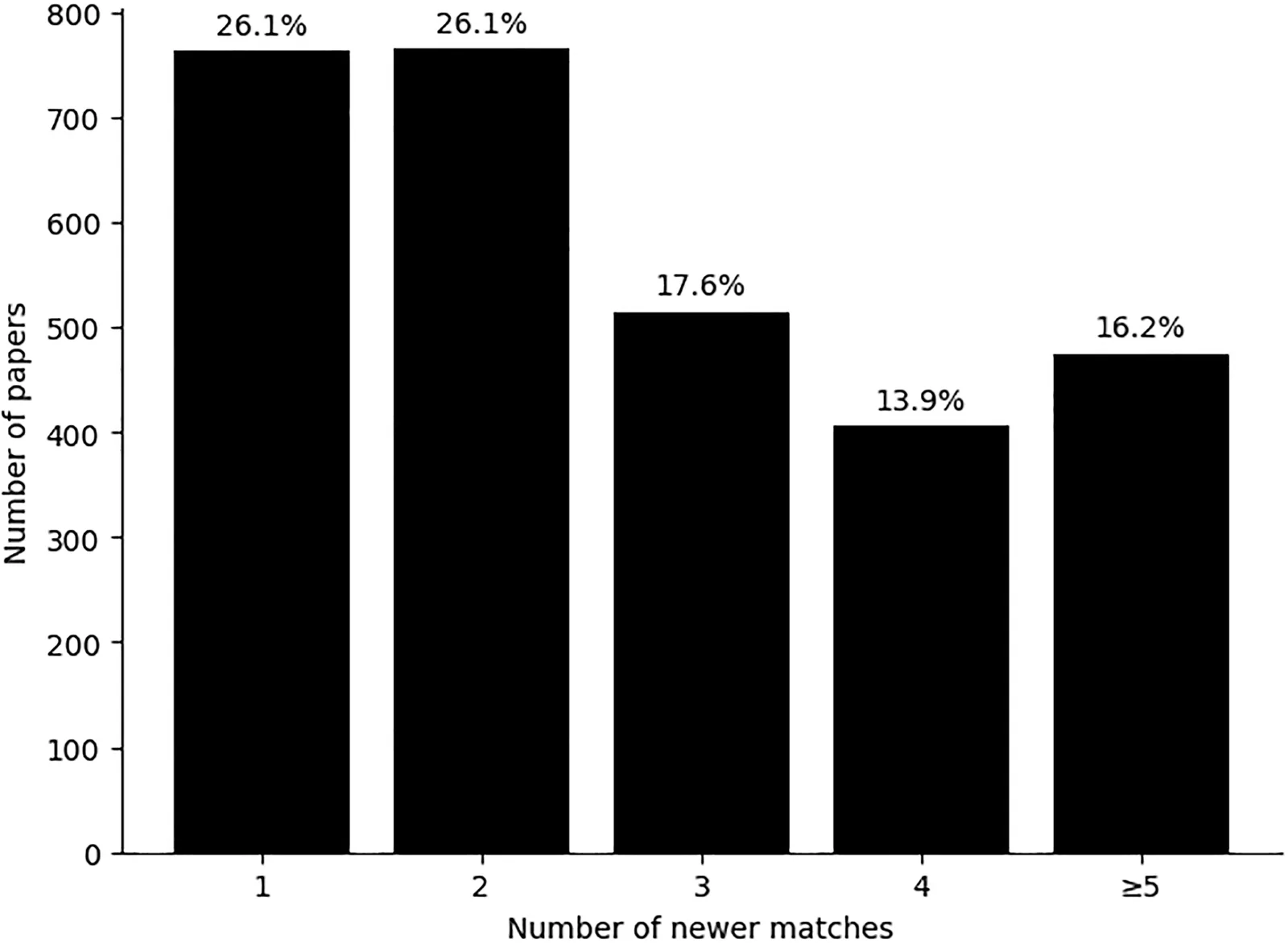
Newer research alternatives available for policy-cited studies Bars show the number of policy-cited papers that had a given number of substantively similar studies published after the cited paper but before the policy document was published.

Figure 1 summarises the number of newer semantic matches per policy-cited article. Across all matched papers, the mean number of post-2020 matches was 3.6. Matches were broadly distributed, with roughly one quarter of cited papers linked to a single newer study and over 15% linked to five or more.

**Figure 2:**
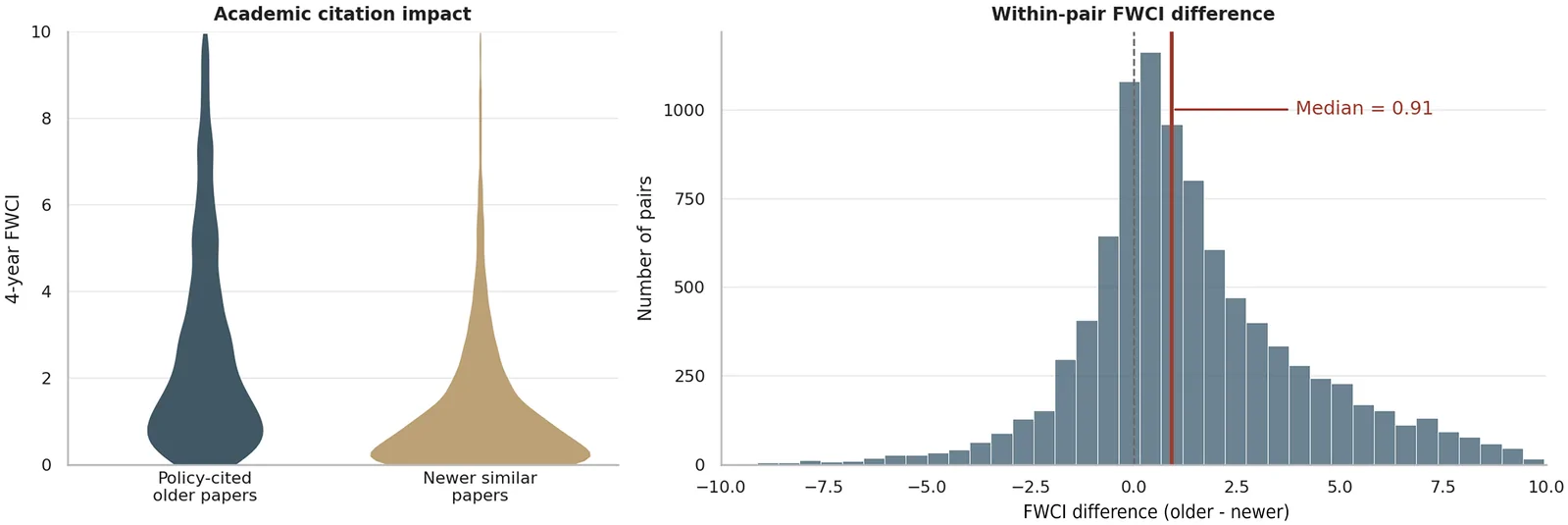
Citation impact of policy-cited papers and their newer semantically similar counterparts (Left) Distribution of Field-Weighted Citation Impact (FWCI) for policy-cited older papers and matched newer papers. (Right) Distribution of within-pair FWCI differences, calculated as older minus newer. Positive values indicate higher citation impact for the cited paper. The median difference is 0.91.

We next compared older cited papers with their newer semantically similar counterparts across indicators associated with research visibility and policy uptake. Older cited papers had substantially higher Field-Weighted Citation Impact (FWCI) than their newer matches (Figure 2). The within-pair distribution is shifted to the right, indicating that cited papers usually had higher citation impact than their newer alternatives. The median FWCI difference was 0.91.

Older cited papers also appeared in journals with slightly higher CiteScore values, although the effect was modest, with a median within-pair difference of 0.2 and a mean difference of 0.44. By contrast, first-author h-index showed no systematic difference between cited papers and their newer semantically similar counterparts, with the within-pair distribution centred around zero.

**Figure 3:**
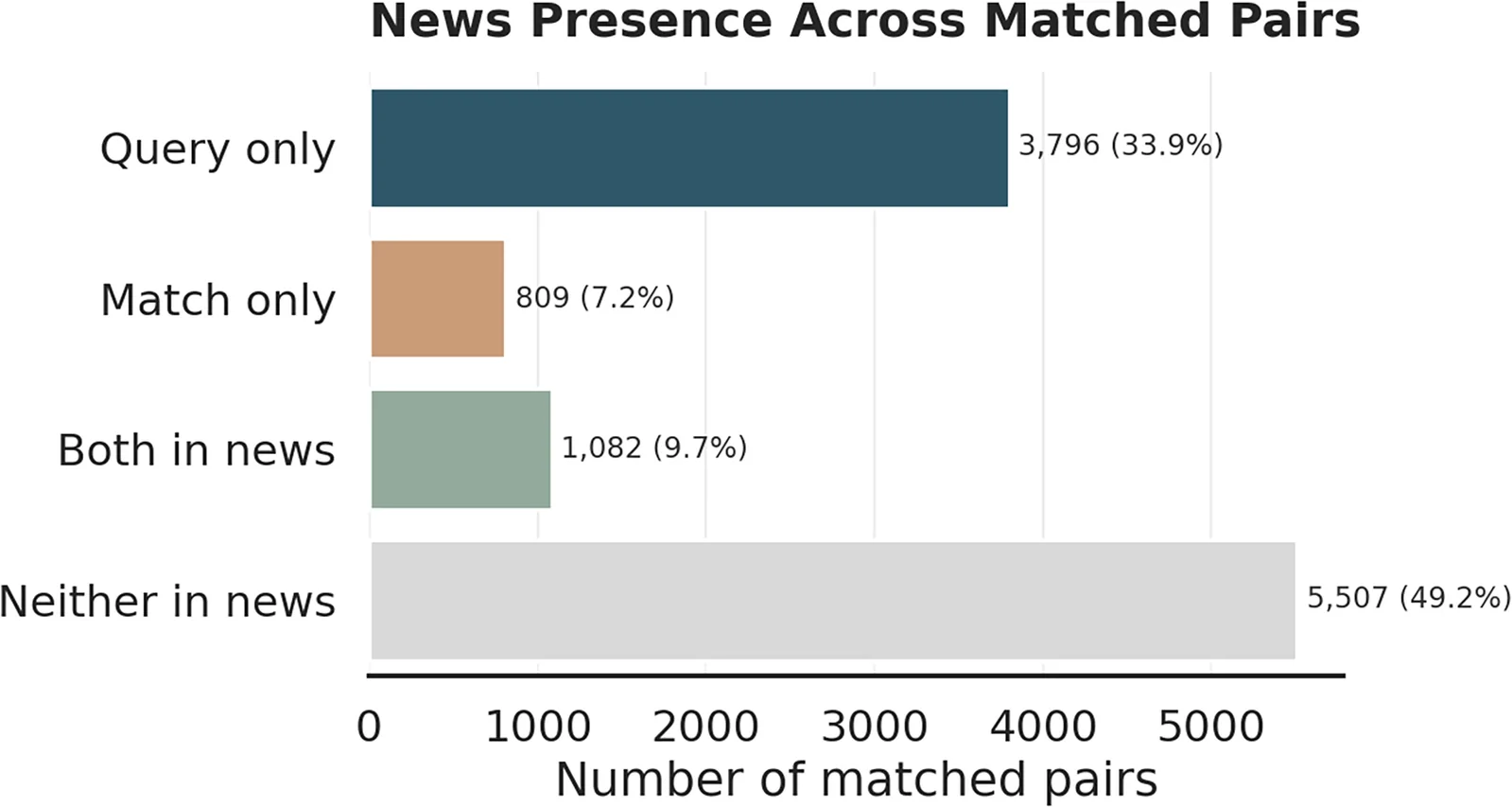
News presence across matched pairs of policy-cited papers and newer semantically similar counterparts Bars show the number of pairs in which only the cited paper, only the matched paper, both papers, or neither paper received news mentions.

News mentions were sparse across the matched pairs (Figure 3). Nearly half of all pairs (49.2%) had no mentions for either paper. However, when mentions were present, they were more often associated with the policy-cited paper. In 33.9% of pairs, only the cited paper received news coverage, compared with 7.2% where only the newer matched paper did. A further 9.7% of pairs had news coverage for both papers.

Finally, we examined whether policy-cited papers that could be semantically matched differed from those that could not. Across FWCI, CiteScore, first-author h-index, and mentions, matched and unmatched papers were broadly similar. Papers without matches had marginally higher average values across most indicators, but median values were similar, suggesting limited systematic differences in impact, journal prestige, author seniority, or media attention.

**Figure 4:**
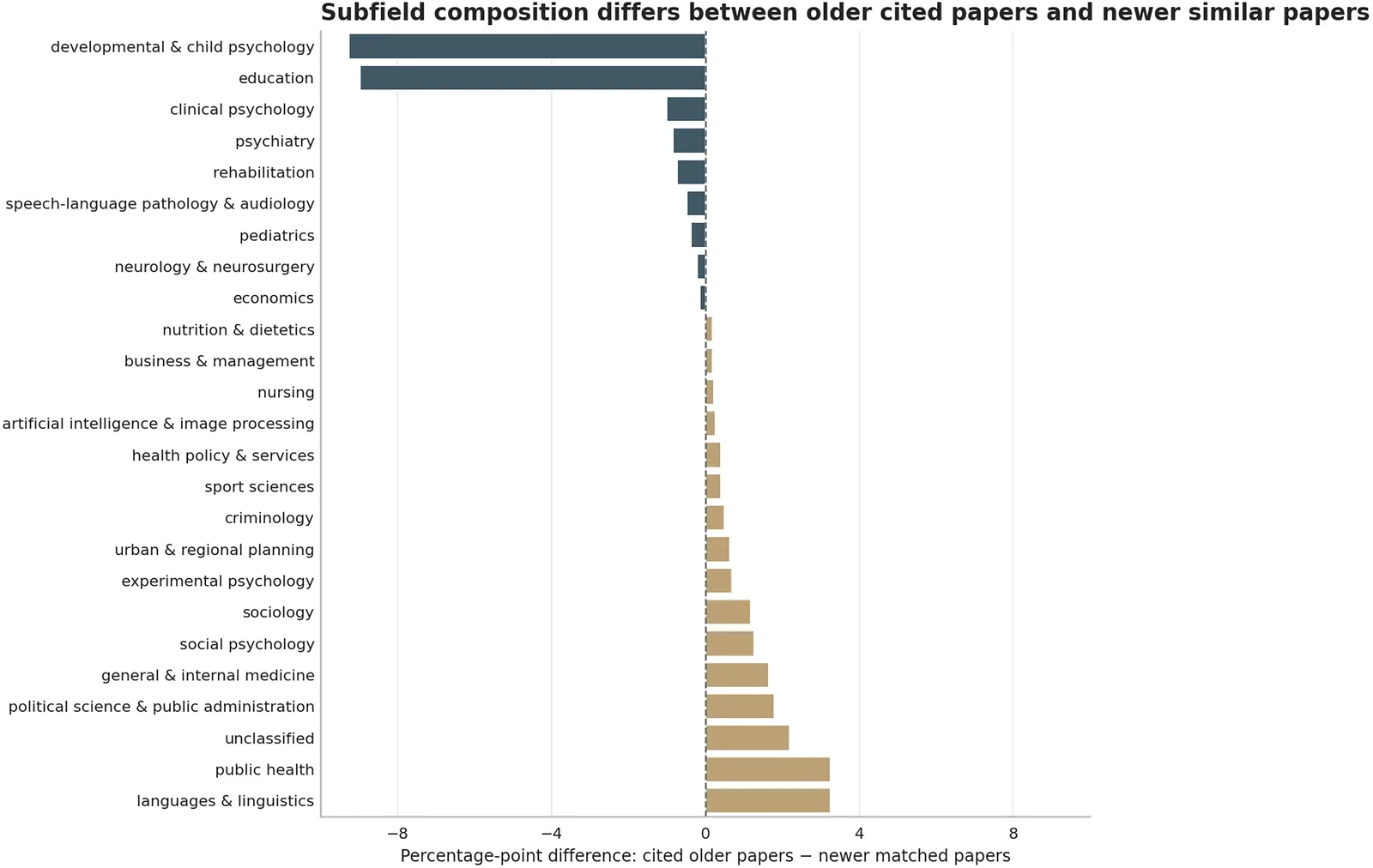
Subfield composition differences between policy-cited papers that could be semantically matched and those that could not Bars show percentage-point differences in subfield representation between matched and unmatched groups.

Subfield composition differed more clearly between the two groups (Figure 4). Papers that could be matched were more concentrated in education and developmental psychology. Papers without matches were more prevalent in fields such as public health, economics, and linguistics. This suggests that the likelihood of identifying newer semantically similar research varies by domain, reflecting differences in research volume, topical structure, and publication dynamics.

## Discussion

Taken together, these results show that for more than half of the pre-2020 studies cited in education policy documents, at least one closely related post-2020 paper was already available when the policy document was published, and most had several. The continued citation of older research therefore often occurred alongside the availability of newer relevant evidence rather than in its absence.

These findings do not imply that newer papers were equivalent substitutes for the older studies cited in policy. Older cited papers tended to have higher citation impact and greater media visibility, and a modest advantage in journal CiteScore, while first-author h-index showed no systematic difference. This suggests that policy reliance on older research may reflect accumulated scientific and public visibility as well as timing.

The results suggest a lag between the production of new research and its incorporation into policy documents. Even in a context characterised by intense scientific activity and strong policy attention, policy documents frequently cited older studies despite the availability of substantively similar and more recent research. This indicates that the presence of relevant research does not guarantee its incorporation into policy outputs. Instead, the findings point to a mismatch between the pace of scientific publication and the pace at which policy systems update the research evidence they draw upon.

Such selectivity reflects path dependence within evidence-use systems. Advisory routines and established expert networks influence which research becomes visible and usable, independent of the volume of newly published work. From this perspective, the findings are best interpreted as evidence of update latency rather than simple non-use of research. Policy systems appear to update their evidentiary bases more slowly than the pace of academic publication even when substantively similar studies are available.

Beyond the COVID-19 education case, the semantic matching approach provides a scalable diagnostic for assessing policy responsiveness to new evidence. Because the analysis anchors on research that policy systems demonstrably used and then measures whether newer comparable studies existed at the time, it allows responsiveness to be evaluated while holding content relevance constant. This structure enables comparative analysis across policy domains, institutions, or jurisdictions, providing a common benchmark for assessing how quickly evidence-use systems incorporate newly available research.

### Limitations

Several limitations define the scope of the inferences drawn from this analysis. First, the study examines a specific subset of policy documents. Although the Overton database indexes a large number of education policy documents produced during the COVID-19 pandemic, coverage is not exhaustive. Moreover, most policy documents do not cite peer-reviewed research. The analysis therefore focuses on the subset of policy outputs that explicitly reference scholarly literature and provides insight into how academic research is incorporated when citations occur rather than a comprehensive account of evidence use in education policymaking.

Second, bibliographic coverage further restricts the analysable corpus. More than 66% of references identified in policy documents could not be linked to Scopus records and were therefore excluded from the semantic matching analysis, and so the method only captures a subset of evidence used, which may be biased due to Scopus indexing criteria.

Finally, the semantic matching approach has methodological limits. Abstract-level embeddings capture similarity in topics and research questions but cannot account for all dimensions that may matter for policy use, such as methodological design, contextual fit, or data quality. The MiniLM model was selected for scalability and cross-domain robustness, but it is not domain-specific and may miss distinctions that are meaningful in particular policy contexts. High semantic similarity should therefore be interpreted as indicating substantive relatedness rather than equivalence and substitution between studies.

## Resource availability

### Lead contact

Further information and requests should be directed to the lead contact, Basil Mahfouz (basil.mahfouz@ucl.ac.uk).

### Materials availability

This study did not generate new unique reagents or materials.

### Data and code availability

The datasets generated for this study are available via GitHub on https://github.com/Coriander89/covid-education-policy-evidence and consist of DOI-to-DOI semantic similarity matches between policy-cited education research and newer related studies and relevant metadata.

## Acknowledgments

We acknowledge Elsevier for funding, technical support, data access, and computational resources. We thank Dr. Andrew Plume for strategic guidance and facilitating access to necessary resources, Kristy James for technical expertise and methodological input, and Alick Bird for infrastructure implementation.

## Author contributions

B.M., L.C., and G.M. conceived the study. B.M. and L.C. developed the methodology. B.M. performed software implementation, data curation, and visualization. B.M. conducted the formal analysis with input from L.C. and G.M. B.M. wrote the original draft of the manuscript. B.M., L.C., and G.M. reviewed and edited the manuscript. L.C. and G.M. supervised the research. G.M. acquired funding.

## Declaration of interests

The authors declare no competing interests.

## Declaration of generative AI and AI-assisted technologies in the writing process

During the preparation of this work, the authors used ChatGPT to assist with language editing, manuscript formatting, and reference formatting. After using this tool, the authors reviewed and edited the content as needed and take full responsibility for the content of the published article.

## STAR★Methods

This study applies the large-scale semantic matching framework developed by Mahfouz et al.^19^ The framework leverages pretrained language models to identify semantically similar research articles across large scale bibliometric databases.

### Key resources table

**Table:** 

REAGENT or RESOURCE	SOURCE	IDENTIFIER
Software and algorithms		
Overton policy database	Overton	https://www.overton.io
Scopus database	Elsevier	https://www.scopus.com or upon request
MiniLM model	Wang et al.^23^	HuggingFace
Elasticsearch	Elastic	https://www.elastic.co
SciSciNet dataset	Lin et al.^24^	https://sciscinet.org

### Experimental model and study participant details

Not applicable. This study uses computational analysis of scholarly publications and does not involve human participants, animals, or biological material.

### Method details

#### Identification of policy-cited research

The aim of this step is to identify scholarly research cited in COVID-19 education policy documents. Policy documents were retrieved from the Overton policy database, which indexes policy publications produced by local, national, and international government bodies and their scholarly ref.^25^

The query retrieved policy documents containing the keywords “COVID-19,” “SARS-CoV-2,” or “coronavirus”. Results were restricted to documents created by government sources, published between March 1, 2020 and May 31, 2023, and classified within education-related subject areas. Specifically, documents were limited to those tagged with the subject area “school” and linked to Sustainable Development Goal 4 (Quality Education). To focus the analysis on policy documents that engage directly with academic research, only documents containing scholarly references were retained.

From the retrieved policy documents, all scholarly references were extracted. The DOIs of cited papers were then matched against the Scopus database to retrieve bibliographic metadata, including publication year and abstract text. These abstracts were used in subsequent steps to generate semantic embeddings for similarity analysis.

Note that Overton is a rapidly expanding policy database. As a result, repeating the same query at a later date may yield a larger set of policy documents. In addition, the methodology used by Overton to classify documents by Sustainable Development Goals has evolved since the time the search for this study was conducted.

#### Embedding generation

Abstracts for research papers published since 2017 were retrieved from Scopus and encoded using the MiniLM (all-MiniLM-L6-v2) transformer model.^23^ MiniLM represents each abstract as a dense vector embedding that encodes the semantic content of the document.

Embedding generation and data access in this study were conducted through a research collaboration with Elsevier, which provided access to Scopus data and computational infrastructure within a unified research platform. Researchers interested in accessing this infrastructure for similar studies may contact the Elsevier Research Collaboration Unit (Dr. Andrew Plume; a.plume@elsevier.com).

Replication is also possible using open-access resources. The SciSciNet dataset provides precomputed SPECTER2 embeddings for large scholarly corpora.^26^ Alternatively, researchers can generate their own embeddings using publication abstracts from OpenAlex, SciSciNet,^24^ or similar bibliographic databases with a transformer-based language model of their choice.

All embeddings were generated from English-language abstracts. This is ensured by Scopus indexing requirements, which mandate that all publications include an English abstract, even when the full text is in another language. When using alternative data sources, language heterogeneity should be considered, as it may affect embedding-based similarity measures and the suitability of the chosen language model.

#### Semantic similarity search

For each policy-cited article published before 2020, the MiniLM embedding of the abstract was used as a query vector and searched against the Scopus corpus to identify semantically similar research papers. Nearest-neighbour retrieval was performed using Elasticsearch vector search, which supports efficient k-nearest-neighbour retrieval across large document collections.

Candidate matches were restricted to papers published between March 2020 and at least three months prior to the publication date of the corresponding policy document. Only papers with a cosine similarity score of 0.8 or higher were retained.

The retained neighbours represent post-2020 studies that are substantively similar to the cited pre-2020 paper and that were already available when the policy document was issued. These papers therefore constitute plausible newer alternatives that could have informed the same policy discussion. Examples of matched abstracts are provided in supplementary materials (SM1).

### Quantification and statistical analysis

Semantic similarity between publications was measured using cosine similarity between MiniLM vector embeddings. A similarity threshold of 0.8 was used to identify substantively related studies. Descriptive statistics were used to quantify the number of semantically similar post-2020 publications associated with each policy-cited article.

### Additional resources

SciSciNet dataset: https://sciscinet.org.

Overton policy database: https://www.overton.io.

miniLM language model: https://huggingface.co/sentence-transformers/all-MiniLM-L6-v2.

## Supplemental information

Table S1. Examples of cited pre-2020 abstracts and matched post-2020 abstracts
