# Supplementary material for: COVID-19 education policy often relied on older research despite available new evidence
Source: STAR Protoc. 2026 Jul 24;7(3):104710. doi: 10.1016/j.xpro.2026.104710 (PMC13615485; doi:10.1016/j.xpro.2026.104710)
Supplement: Supplementary file 1 [file mmc1.pdf]

Supplementary Material

Supplementary Table 1 (SM1). Examples of cited pre-2020 abstracts and matched post-2020 abstracts

This table provides illustrative examples of cited pre-2020 abstracts and semantically matched post-2020 abstracts. The examples show how the matching procedure identified newer studies that addressed closely related topics and were therefore plausible newer alternatives to older research cited in policy documents.

Cited Abstract (pre2020)	Matched Abstract (post 2020)
There is a large body of research examining the discipline experiences of Black males (Lewis et al. in Souls: A Critical Journey of Black Politics, Culture, and Society,2009; Skiba et al. in The Urban Review, 34, 317-348, 2002); however, less is known about the types of behavioral infractions Black female students exhibit and the discipline sanctions imposed for Black girls for such infractions. As a result, the purpose of this study is to examine the type of discipline infractions exhibited by Black female students enrolled in an urban school district and to explore whether the pattern of discipline infractions and sanctions imposed for Black girls disproportionately differs from all female students, but more specifically White and Hispanic females. Results suggest that Black girls are overrepresented in exclusionary discipline practices and Black girls reason for discipline referrals differs significantly from White and Hispanic girls. Based on these findings, recommendations are provided for urban educational stakeholders	Studies have documented the ubiquitous racial disparities in school discipline. However, knowledge of the unique and complex disciplinary experiences of Black girls, shared from their own voices, remains under-researched. To better understand the school policing and 'pushout' phenomenon for Black girls, this qualitative study examines data which explore the resulting consequences of a fight between 30 Black female students. Our findings suggest that the hypercriminalization and subsequent punitive measures inflicted on these Black girls hindered their academic experiences and that despite the threat of exclusionary discipline sanctions, these young girls fought–not because they were bad–but out of necessity. Concluding, we discuss restorative approaches and explain how Circles can help support emotional healing and educational reintegration after moments of conflict.
This study reports on relationships among gender, maternal depressed mood, and children's trajectories of depressive phenomena across middle childhood and early adolescence. It tested the hypothesis that, compared to boys, girls become increasingly vulnerable to maternal depression as they enter adolescence. The study sample consisted of 834 families from 10 Pacific Northwest schools that participated in the Raising Healthy Children project. Maternal depressed mood and children's depressive phenomena were assessed annually during an 8-year period that spanned Grade 3 through Grade 10 for the children. Mean scores for girls' depressive phenomena increased relative to those for boys as children matured. Maternal depressed mood was significantly and positively associated with children's level of depressive phenomena. An interaction effect of gender and maternal depressed mood on acceleration in children's depressive phenomena indicated that girls' trajectories of depressive phenomena were sustained in the presence of maternal depression while those of boys declined in the presence of maternal depression.	Maternal depression has consistently demonstrated reliability as a predictor of major depression in children. However, the effect of gender as a potential moderator remains under-researched. This study examined gender differences in depression and hypothesized that maternal depression would be more greatly associated with depression in girls by early adolescence. A representative sample of 7237 participants from the Growing Up in Ireland national longitudinal study of Irish children was examined across two waves when participants were aged 9 and 13. Separate multiple regression analyses were run for girls and boys to ascertain if maternal depression and other key variables predicted child depression by age 13. A third analysis looked at the effect of gender. Maternal depression when the children were aged 9 was found predictive of depressive symptomology by age 13 in girls but not for boys. Additionally, conflict with caregivers, household income, and bullying were significant predictors for both genders. Gender significantly predicted youth depression when the child was female (R^2 =.13, f^2 =.25). These findings indicate that late childhood to early adolescence is a vulnerable period for girls during which they appear more susceptible to

	developing depressive symptomology than boys, particularly when exposed to maternal depression.
This work interrupts the current "consulting students" discourse that positions students as service clients and thus renders more problematic the concept of student voice in ways that it might be sustained as a democratic process. It looks at student voice holistically across realms of classroom practices, higher education, practitioner inquiry and policy formulation. The authors render problematic the "empowerment" rhetoric that is the dominant and insufficient narrative justifying consulting children and young people. They explore the many contradictions and ambiguities associating with recruiting and encouraging them to participate and the varying impacts of different circumstances on the ways in which student voice projects are enacted. They perceive that it is possible for student voice projects to be subverted from both above and below as varying stakeholders with varying purposes struggle to manage and control projects. Importantly, the book reports on research that identifies and highlights conditions for initiating and sustaining student voice and include "beyond school" dimensions that consider young people as "audiences" who can inform community facilities, their development and design as well as undergraduate students in universities. These cases are not reported as celebratory, but rather act as narratives that illuminate the many challenges facing those who chose to work with young people in authentic ways. It both advances methodologies for engaging young people as active agents in the design and interpretation of research that concerns them and offers a critique of those methods that see young people as the objects of research, where the data is mined for purposes that do not recognise that students are the consequential stakeholders with respect to decisions made in their interests.	This paper makes a novel and important contribution to scholarship by developing and presenting a set of concepts and questions for those researching student voice in Ireland to consider and explore in their studies, and specifically in relation to classroom practice at post-primary level. Here, a distinction is drawn between consultations that take place inside classrooms between students and teachers and consultations that take place between management and students and cognisance is paid to school patronage, school socio-economic context, and the career stage of teachers and positions in the school hierarchy. This paper ultimately offers a heuristic device as a starting point for future research on student voice in Irish post-primary schools and sets out to bring about more critical thinking regarding how student voice plays out vis-à-vis classroom practice.
The role of the principal is imperative in creating a school climate that nurtures differences and equality at the same time. Policy and legislation in Ireland dictates the responsibility of school principals in preventing and intervening in issues relating to bullying. The current research investigates the extent to which principals consider the impact of bullying on their students and the strategies they take to combat it in their schools. We consider how principals respond to bullying and what supports they feel are missing for them to adhere to relevant policy. A sample of 918 principals from a range of schools across Ireland completed an online survey. The results demonstrated some positive actions by principals such as the provision of anti-bullying policies. However, they also document areas that need more action such as increased access to counsellors and appointing a specific staff member to deal with and tackle bullying in each school. Guidance from the Department of Education and Skills in Ireland on the development and provision of	This study aimed to investigate how public school teachers perceive student bullying and how they perceive their own as well as school administrators' efforts to protect students from bullying. A total of 238 elementary, middle, and high school teachers responded to 18 questions. The study found that student bullying exists in Kuwait public schools, it is more widespread in boys' schools, and it occurs more in middle schools. Further, the study found a significant relationship between student bullying and school administrators' efforts to protect students from bullying. A discussion of these results and relevant recommendations for future studies are included.

an anti-bullying programme is called for. Results are discussed in light of the current Anti-Bullying Procedures established by the Department of Education and Skills and the Action Plan for Bullying 2013.	
This study examines the effects of universal Early Childhood Education and Care (ECEC) on child development and children's later life outcomes. Using meta-analytical techniques, we synthesize the findings from recent studies that exploit natural experiments to identify the causal effects of universal ECEC arrangements. We use 250 estimates from 30 studies conducted between 2005 and 2017. Our meta-regressions include estimates on a wide variety of children's outcomes, ranging from (non-)cognitive development measured during early childhood to educational outcomes and earnings in adulthood. Overall, the evidence on universal ECEC is mixed. Age of enrollment is not a major factor in explaining the impact. Some evidence indicates that more intensive programs produce more favorable outcomes. Program quality matters critically: high quality arrangements consistently generate positive child outcomes. Publicly provided programs produce more favorable effects than privately provided (and mixed) programs. There is no evidence of fading out. Furthermore, the gains of ECEC are concentrated within children from lower socioeconomic families.	Objective: This study investigates some of the mechanisms, which may explain social inequalities in the usage of early childhood education and care (ECEC) by focusing on a recently born child cohort born in Germany, a universal childcare regime. Background: Research recognizes rational cost–benefit considerations as important for understanding social inequalities in educational decisions. Yet, given data limitations, we know relatively little about how these considerations are associated with inequalities in ECEC take-up. Thus, we model the decision to use ECEC as a rational cost–benefit investment strategy, which simultaneously affects the human capital of mothers and children. Method: We test our assumptions with data from the newborn cohort of the National Educational Panel Study (NEPS), estimating the timing of first entry into ECEC, using discrete-time event history models (N = 3257). Results: Results indicate that cost–benefit calculations are relevant for the timing of ECEC take-up, but do not explain social differences. Mothers who perceive ECEC as an investment in child development, and as an opportunity to maintain their own employment status use ECEC earlier. This association is particularly pronounced for more highly educated mothers. Conclusion: Findings highlight mothers' awareness of future educational returns for ECEC decisions. Additionally, they illustrate the importance of the cultural and structural context for these decisions. © 2022 The Authors. Journal of Marriage and Family published by Wiley Periodicals LLC on behalf of National Council on Family Relations.
